# Inhibition of SQSTM1 S403 phosphorylation facilitates the aggresome formation of ubiquitinated proteins during proteasome dysfunction

**DOI:** 10.1186/s11658-023-00500-6

**Published:** 2023-10-24

**Authors:** Chenliang Zhang, YiChun Duan, Chen Huang, Liping Li

**Affiliations:** 1 Division of Abdominal Tumor Multimodality Treatment, Cancer Center and Laboratory of Molecular Targeted Therapy in Oncology, West China Hospital, Sichuan University, Chengdu, 610041 Sichuan Province China; 2 Division of Abdominal Tumor Multimodality Treatment, Cancer Center, West China Hospital, Sichuan University, Chengdu, 610041 Sichuan Province China; 3 Department of Pharmacy, Chengdu Fifth People’s Hospital, Chengdu University of Traditional Chinese Medicine, Chengdu, 611130 Sichuan Province China

**Keywords:** SQSTM1 phosphorylation, Proteasome inhibition, Aggresome, Cell death, Aggrephagy

## Abstract

**Background:**

Ubiquitin–proteasome-system-mediated clearance of misfolded proteins is essential for cells to maintain proteostasis and reduce the proteotoxicity caused by these aberrant proteins. When proteasome activity is inadequate, ubiquitinated proteins are sorted into perinuclear aggresomes, which is a significant defense mechanism employed by cells to combat insufficient proteasome activity, hence mitigating the proteotoxic crisis. It has been demonstrated that phosphorylation of SQSTM1 is crucial in regulating misfolded protein aggregation and autophagic degradation. Although SQSTM1 S403 phosphorylation is essential for the autophagic degradation of ubiquitinated proteins, its significance in proteasome inhibition-induced aggresome formation is yet unknown. Herein, we investigated the influence of SQSTM1 S403 phosphorylation on the aggresome production of ubiquitinated proteins during proteasome suppression.

**Methods:**

We examined the phosphorylation levels of SQSTM1 S403 or T269/S272 in cells after treated with proteasome inhibitors or/and autophagy inhibitors, by western blot and immunofluorescence. We detected the accumulation and aggresome formation of ubiquitinated misfolded proteins in cells treated with proteasome inhibition by western blot and immunofluorescence. Furthermore, we used SQSTM1 phosphorylation-associated kinase inhibitors and mutant constructs to confirm the regulation of different SQSTM1 phosphorylation in aggresome formation. We examined the cell viability using CCK-8 assay.

**Results:**

Herein, we ascertained that phosphorylation of SQSTM1 S403 did not enhance the autophagic degradation of ubiquitinated proteins during proteasome inhibition. Proteasome inhibition suppresses the phosphorylation of SQSTM1 S403, which facilitated the aggresome production of polyubiquitinated proteins. Interestingly, we found proteasome inhibition-induced SQSTM1 T269/S272 phosphorylation inhibits the S403 phosphorylation. Suppressing S403 phosphorylation rescues the defective aggresome formation and protects cells from cell death caused by unphosphorylated SQSTM1 (T269/S272).

**Conclusions:**

This study shows that inhibition of SQSTM1 S403 phosphorylation facilitates the aggresome formation of ubiquitinated proteins during proteasome dysfunction. SQSTM1 T269/S272 phosphorylation inhibits the S403 phosphorylation, boosting the aggresome formation of ubiquitinated protein and shielding cells from proteotoxic crisis.

**Supplementary Information:**

The online version contains supplementary material available at 10.1186/s11658-023-00500-6.

## Introduction

Removing misfolded proteins by the ubiquitin–proteasome-system (UPS) is essential for cells to maintain proteostasis and reduce the proteotoxicity caused by these aberrant proteins [1, 2]. When proteasome activity is inadequate, ubiquitinated proteins are sorted into perinuclear aggresomes [3, 4]. Numerous investigations have established that the aggresome formation mediated by proteasome inhibition facilitates the segregation of toxic misfolded proteins, hence mitigating the proteotoxic crisis [3–7].

Aggresome formation is a complex biological process governed by numerous functional factors [8]. Although the molecular mechanism is not entirely obvious, multiple studies have revealed that aggresome formation in cultured cells treated with proteasome inhibitors involves two steps. First, upon proteasome inhibition, ubiquitinated misfolded proteins are organized into micro-aggregates, where molecular chaperones, such as heat shock protein 70 (HSP70), E3 ubiquitin ligase, like carboxyl-terminal of Hsp70/Hsp90 interacting protein (CHIP), and co-chaperones, like Bcl-2-associated athanogene 3 (BAG3), play a crucial role [9–12]. Our earlier study reported that the M2 isoform of pyruvate kinase (PKM2) is also implicated in the CHIP-HSP70-BAG3 complex-mediated ubiquitinated protein aggregation [13]. Second, with functional proteins, like HDAC6 and Dynein, micro-aggregates are swiftly carried along the microtubules to the microtubule organizing center (MTOC), ultimately forming a higher aggregation structure-aggresome [4, 14–16]. Although under numerous cellular stresses, such as endoplasmic reticulum (ER) stress and oxidative stress, misfolded proteins aggregates help their elimination by selective autophagy (aggrephagy) [17–19], aggrephagy's function in cells with inhibited proteasome activity remains disputed. Our previous studies have shown that during proteasome inhibition suppressing the aggrephagy can increase the aggresome formation of ubiquitinated proteins and prevent cell damage [20].

As multifunctional scaffold proteins, SQSTM1 is a key regulator of aggrephagy and aggresome formation [21, 22]. SQSTM1 possesses a C-terminal ubiquitin association (UBA) domain that can bind to polyubiquitinated proteins and a microtubule-associated protein light chain 3 (LC3)-interacting region (LIR) domain that mediates the interaction between SQSTM1 and LC3/Atg8, an essential autophagosome membrane protein [23]. As an autophagy receptor, these functional domains permit SQSTM1 to sequester polyubiquitinated cargos within autophagosomes for destruction by the autophagy-lysosomal system. Moreover, the N-terminal Phox and Bem1 (PB1) domains of SQSTM1 not only cause oligomerization of SQSTM1 and aggregation of ubiquitinated proteins, but also present ubiquitinated proteins to the proteasome for degradation by binding to the proteasome subunits [24]. Among the multiple mechanisms involved in aggrephagy and aggresome formation, phosphorylation alteration is crucial to transforming SQSTM1's regulatory roles. The SQSTM1 phosphorylation at S403 in the UBA domain by casein kinase 2 (CK2), TANK-binding kinase 1 (TBK1), and UNC-51-like kinase 1 (ULK1) increases its affinity for ubiquitinated proteins and promotes their destruction by autophagy [25–27]. Moreover, during proteasome inhibition, Pink1-s (short form of PTEN-induced putative kinase 1) mediated the phosphorylation of SQSTM1 at S28 can promote the segregation of ubiquitinated proteins into aggresome [9]. Recently, we have reported that proteasome inhibition can trigger p38γ/δ-mediated phosphorylation of SQSTM1 at T269/S272, which limits the autophagic sequestration of SQSTM1 and promotes the formation of ubiquitinated protein-associated aggresome [6, 20].

In this report, we examined the influence of SQSTM1 S403 phosphorylation on the aggresome production of ubiquitinated proteins during proteasome suppression. We demonstrated that phosphorylation of S403 inhibits the proteasome inhibitor-induced aggresome production of polyubiquitinated proteins. T269/S272 phosphorylation in SQSTM1 enhances aggresome formation by inhibiting S403 phosphorylation. Our findings indicate a crosstalk mechanism between SQSTM1 S403 and T269/S272 phosphorylation during ubiquitinated protein-associated aggrephagy and aggresome formation.

## Methods

### Chemical reagents

MG132 (Merck Millipore, 474790); Bafilomycin A1 (CSNpharm, CSN10374); Bortezomib (Selleck, S1013); CX-4945 (MCE, HY-HY-50855); BX795 (MCE, HY-10514); SBI-0206965 (CSNpharm, CSN16884); Doramapimod (CSNpharm, CSN10856); Wortmannin (MCE, HY-10197).

### Plasmid construction

pcDNA3.1-SQSTM1 (WT, T269A/S272A, T269E/S272D), pcDNA3.1-FLAG-p38δ (K54R, F324S) were constructed as described previously [6]. For generation of SQSTM1 (S403A, S403E, T269A/S272A/S403A) and p38γ (K56R, D179A) expression vectors, the ORF Fragments were amplified by PCR from the genes and cloned into pcDNA3.1 with FLAG tag using Uniclone One Step Seamless Cloning Kit (GeneSand, Beijing). All the plasmids were verified by DNA sequencing. Primer sequence information used for ORF amplification is listed in Additional file 1: Table S1.

### Cell culture and transfection

Wild-type AD293, SQSTM1 knockout AD293, and SQSTM1 re-expressing cell lines were obtained and constructed as described in previous studies [6, 20]. Hela cells and A375 cells were purchased from ATCC. HEK293FT cell line was purchased from Thermo Fisher Scientific and used for lentivirus production. All cells were cultured at 37 ℃ in Dulbecco’s modified Eagle’s medium (DMEM, Gibco, 12800082) supplemented with 10% fetal bovine serum (FBS, HyClone, SH30071.03), 1% penicillin–streptomycin (Thermo Fisher Scientific, 15140211), 1 × non-essential amino acids (NEAAs, Thermo Fisher Scientific, 11140076) and 2 mM l-glutamine (Thermo Fisher Scientific, 25030081) in a 5% CO_2_ incubator. All the cell lines were authenticated and tested for contamination. Plasmid transfection was carried out with Megatran (OriGene, TT200003) following the manufacturer's instructions.

### Generation of stable expression cell lines

To generate stably re-expressed wildtype or mutated SQSTM1 cell lines, the DNA fragments corresponding to the ORFs of SQSTM1 were cloned into lentiviral vectors, then transfected into HEK293FT cells with helper plasmids to package lentiviral. Stable cell selection was carried out as described previously [6].

### Protein extraction and western blot analysis

The total protein extract was prepared by homogenizing the cells in 1 × SDS sample buffer. All the protein samples were boiled for 5 min and then resolved in SDS-PAGE. The protein samples separated by SDS-PAGE were transferred to the Immunobilon-FL PVDF membrane (Merck Millipore, IPFL00010). After blocking with 5% non-fat milk in TBST (TBS, pH 7.4, 0.1% Tween-20), the membrane was incubated with primary antibody at 4 ℃ overnight. The membrane was washed and incubated with secondary antibodies in the dark for 2 h. The image was acquired by Li-Cor Odyssey Clx Infrared Imaging System (LI-COR Biotechnology, Lincoln, NE, USA). The following primary and secondary antibodies were used for western blotting assay: SQSTM1 (Proteintech, 18420-1-AP), ubiquitin (Abcam, ab134953); Phospho-SQSTM1 (Thr269/Ser272)-specific antibody (Phosphosolutions, P196-269); Phospho-SQSTM1 (S403)-specific antibody (Cell Signaling Technology, 39786); FLAG tag (Prospec, ANT-146); GAPDH (Zen Bioscience, 200306); β-actin (Zen Bioscience, 200068-6D7). Dylight 680, or Dylight 800-conjugated secondary antibodies (Thermo Fisher Scientific, A28183, A27042, 35518). See Additional file 1: Table S2 for further details and dilutions of all antibodies.

### Immunostaining

Immunostaining was performed as described previously [12]. In brief, after treatment, cells were fixed with 4% paraformaldehyde in PBS for 15 min, permeabilized for 15 min with 0.2% Triton X-100 (Merck Millipore, 94101-L), and then blocked with 5% goat serum for 1 h. they were subsequently incubated with primary antibodies overnight at 4 °C followed by incubation for 1 h with secondary antibodies at room temperature. Their nuclei were stained with a DAPI solution. Images were captured with a fluorescent microscope (Nikon Eclipse 80i equipped with Nikon PLAN FLUOR × 40 objective) or Nikon confocal microscope (Nikon, N-STORM and A1). Photographic images were resized and analyzed by ImageJ software. The following primary antibodies were used for immunostaining: SQSTM1 (Santa Cruz Biotechnology, sc-28359); K48-linked Ub chain-specific antibody (Merck Millipore, 05-1307); FLAG Tag (Prospec, ANT-146); Phospho-SQSTM1 (S403)-specific antibody (Cell Signaling Technology, 39786), Alexa Flour 488- or 568-conjugated secondary antibodies (Thermo Fisher Scientific, A11034, A11029, A11031, A11036); See Additional file 1: Table S2 for further details and dilutions of all antibodies.

### Cell viability assay

Cells were seeded into 96-well plates. After treated with the indicated reagent, the cell viability was determined by Cell Counting-8 Kit (Dojindo, Kumamoto, Japan) as recommended by the manufacturers. The efficacy of drugs on cell growth was normalized to untreated control.

### Statistical analyses

Each experiment was performed at least three times. Western blot was measured by ImageJ. Fluorescence images from aggresome formation results were quantified manually. Data were represented as mean ± SEM. Comparisons between individual data points were made using a two-tailed Student *t* test (2 groups) or one-way ANOVA analysis (> 2 groups). Differences were considered statistically significant when *P* < 0.05. All statistical analyses were performed using Graph Prim 7.0.

## Results

### Proteasome inhibition suppresses the phosphorylation of SQSTM1 S403

**Fig. 1:**
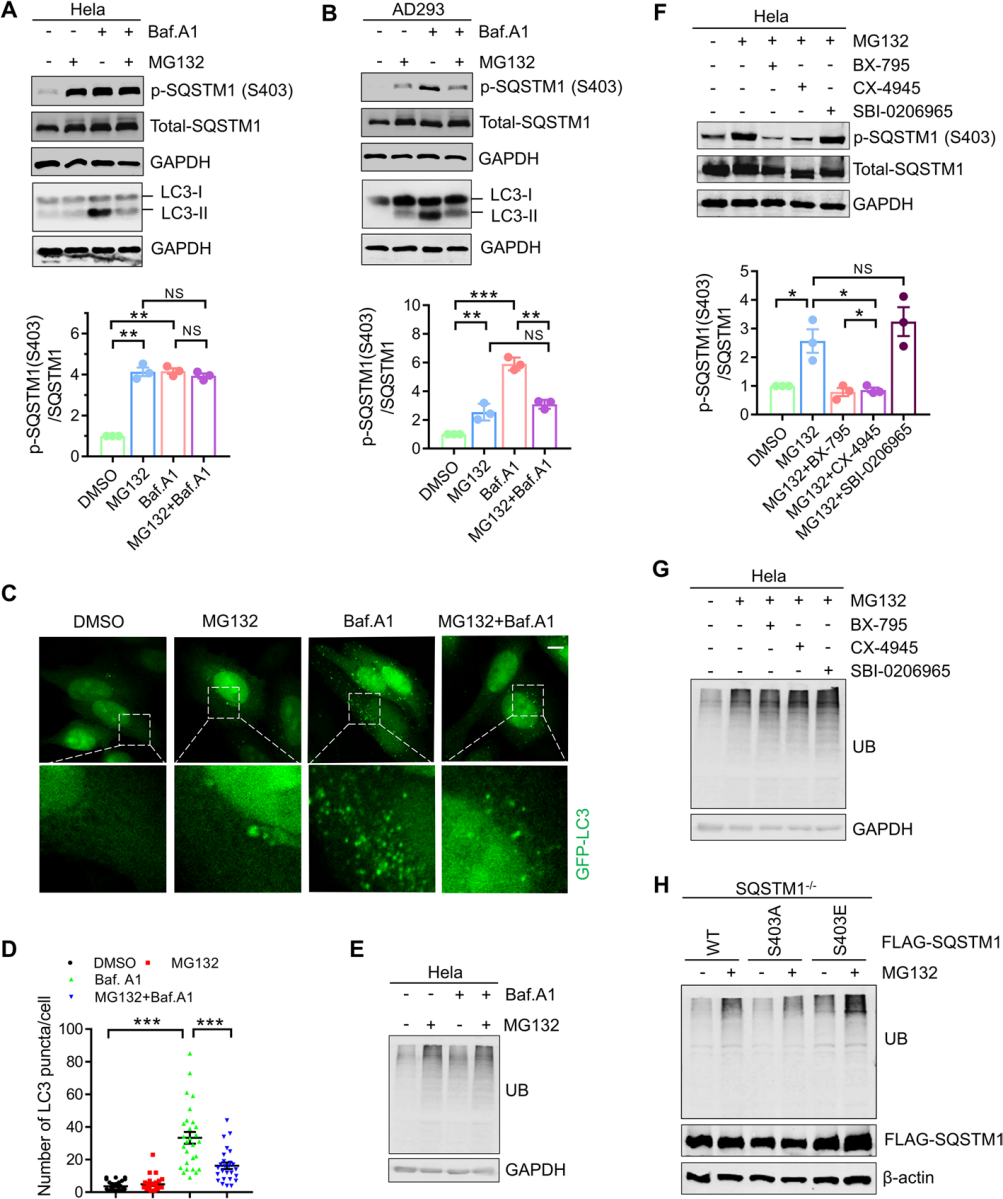
SQSTM1 S403 phosphorylation does not affect the autophagic degradation of ubiquitinated proteins during proteasome inhibition. **A–B** Hela cells and AD293 cells were treated with MG132 (2 μM) and Bafilomycin A1 (25 nM), alone or in combination for 14 h. The whole-cell lysates were subjected to western blot analysis with indicated antibodies. Data are mean ± SEM of three independent experiments; **P < 0.01, ***P < 0.001, *NS* no significance. **C** Hela cells stably expressing GFP-LC3 were treated with MG132 (1 μM) and Bafilomycin A1 (25 nM), alone or in combination for 14 h. The cells were fixed, the images were captured with confocal microscope. Scale bar: 10 μm. **D** Quantitative analysis of results in **C**. About 25 cells from three independent experiments were scored for each group. Data are mean ± SEM; ***P ≤ 0.001. **E** Hela cells were treated with MG132 (2 μM) and Bafilomycin A1 (25 nM), alone or in combination for 14 h. The whole-cell lysates were subjected to western blot analysis with indicated antibodies. **F**-**G** Hela cells were treated with MG132 (2 μM) alone, or combined with BX-795 (5 μM)/CX-4945 (10 μM)/SBI-0206965 (10 μM) for 14 h. The whole-cell lysates were subjected to western blot analysis with indicated antibodies. Data are mean ± SEM of three independent experiments; **P < 0.01, ***P < 0.001, *NS* no significance. **H** SQSTM1 knockout AD293 cells stably expressing indicated constructs were treated with or without MG132 (2 μM) for 14 h. The whole-cell lysates were subjected to western blot analysis with indicated antibodies

Since SQSTM1 S403 phosphorylation is essential for the aggrephagy of ubiquitinated proteins [25], we studied the influence of proteasome inhibition on SQSTM1 S403 phosphorylation. We utilized the proteasome inhibitor MG132 and the autophagic flux inhibitor Bafilomycin A1 to reduce proteasome activity and block autophagic flux, either alone or in combination and measured the phosphorylation level of SQSTM1 S403. As depicted in Fig. 1 A, we discovered that in Hela cells, both suppression of proteasome activity and inhibition of autophagy can induce a substantial rise in SQSTM1 S403 phosphorylation. The combination inhibition of proteasome activity and autophagy had no further effect on the accumulation of S403 phosphorylation as compared to the therapy alone, indicating that inhibition of proteasome and blocking of autophagy may share the same fundamental mechanism for regulating the phosphorylation of SQSTM1 S403. Matsumoto et al. have demonstrated that phosphorylated S403 can be quickly eliminated by the autophagy pathway [25]. Sha and colleagues have reported that the initiation of autophagy is postponed following proteasome inhibition [28]. The aggresome formation of misfolded protein prior to the activation of autophagy emerges as a crucial strategy for cellular defense against proteotoxic crises [28]. As reported in our previous study [20], autophagosome production in cultured cells is inhibited in the early stage of proteasome inhibition (Fig. 1A–D). Therefore, the increase of S403 phosphorylation caused by a proteasome inhibitor may result from autophagy inhibition. Intriguingly, we discovered that inhibiting autophagy in AD293 greatly increased the accumulation of phosphorylated SQSTM1S403 relative to proteasome inhibition. Moreover, we also showed that the proteasome inhibitor can considerably diminish the accumulated S403 phosphorylation caused by autophagy inhibition (Fig. 1B). These data show that Hela cells lack a molecular mechanism that underlies the suppression of SQSTM1 S403 phosphorylation in response to inhibition of the proteasome in AD293 cells.

### SQSTM1 S403 phosphorylation does not affect the autophagic degradation of ubiquitinated proteins during proteasome inhibition

Next, we examined whether the phosphorylation of SQSTM1 S403 influences the autophagic degradation of ubiquitinated proteins during proteasome suppression. As depicted in Fig. 1E, in Hela cells, blocking autophagy did not increase the accumulation of polyubiquitinated proteins compared to inhibiting the proteasome alone. According to previous research, TBK1, CK2, and ULK1 are the primary kinases that phosphorylate SQSTM1 S403 [25–27]. We attempted to suppress SQSTM1 S403 phosphorylation using targeted inhibitors for these kinases. As depicted in Fig. 1F, TBK1 inhibitor BX-795, and CK2 inhibitor CX-4945 could substantially reduce the phosphorylation level of SQSTM1 S403 enhanced by proteasome inhibitors, but not the ULK1 inhibitor, suggesting that ULK1 is not involved in the regulation of SQSTM1 S403 phosphorylation under our treatment conditions. However, we did not observe the further accumulation of ubiquitinated proteins in Hela cells cotreated with CK2 or TBK1 inhibitor and a proteasome inhibitor (Fig. 1G). These results demonstrate that although proteasome inhibitors can significantly increase SQSTM1 S403 phosphorylation, this does not cause the autophagic degradation of ubiquitinated proteins in Hela cells. To validate further the nonfunctional involvement of SQSTM1 S403 phosphorylation in the degradation of ubiquitinated proteins during proteasome inhibition, In SQSTM1 knockout AD293 cells, we re-expressed wildtype SQSTM1, the non-phosphorylatable (S403A) mutant, and the phosphomimetic (S403E) mutant. Then we evaluated the impact of these mutants on ubiquitinated protein clearance. As depicted in Fig. 1H, neither S403A nor S403E mutation significantly altered the number of ubiquitinated proteins during proteasome suppression. These findings suggest that SQSTM1 S403 phosphorylation does not affect the autophagic degradation of ubiquitinated proteins when the proteasome is blocked.

### Phosphorylation of SQSTM1 S403 suppresses the aggresome formation of ubiquitinated proteins during proteasome inhibition

**Fig. 2:**
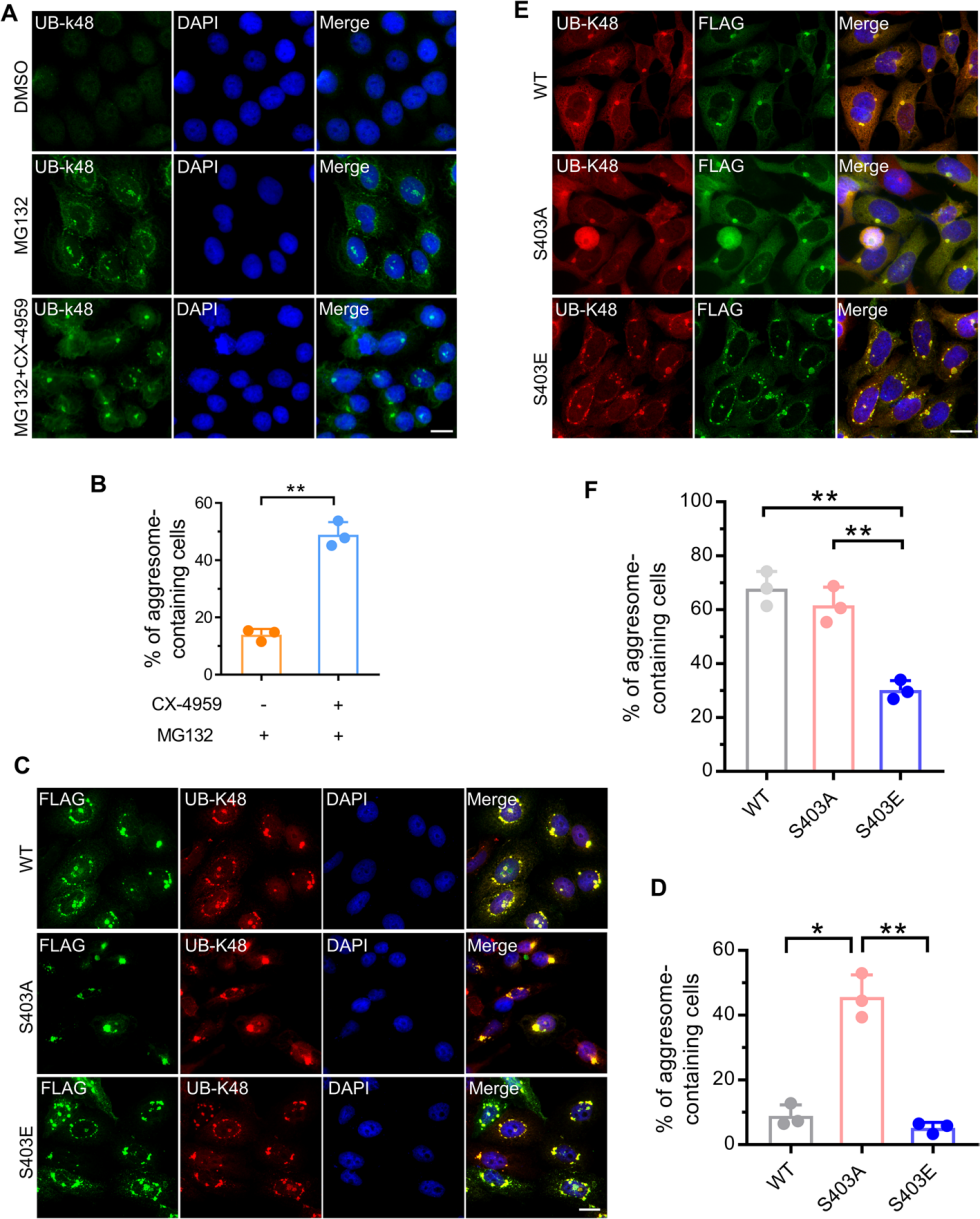
Phosphorylation of SQSTM1 S403 suppresses the aggresome formation of ubiquitinated proteins during proteasome inhibition. **A** Hela cells were treated with MG132 (1 μM) alone or combined with CX-4945 (5 μM) for 14 h. The aggresome formation was analyzed by immunostaining with anti-UB-K48 antibodies. Nuclei were stained with DAPI (blue). Scale bar: 20 μm. **B** Quantitative analysis of results in (**A**). **C** HeLa cells were transfected with plasmids expressing FLAG-SQSTM1 wildtype (WT), FLAG-SQSTM1 (403A), FLAG-SQSTM1 (403E) for 24 h, and then treated with 1 μM MG132 for 14 h. The aggresome formation was analyzed by immunostaining with anti-UB-K48 (Red) and anti-FLAG (Green) antibodies. Nuclei were stained with DAPI (blue). Scale bar: 20 μm. **D** Quantitative analysis of results in **C**. **E** SQSTM1 knockout AD293 cells stably expressing indicated constructs were treated with 1 μM MG132 for 14 h. The aggresome formation was analyzed by immunostaining with anti-UB-K48 (Red) and anti-FLAG (Green) antibodies. Nuclei were stained with DAPI (blue). Scale bar: 20 μm. **F** Quantitative analysis of results in **E**. For **B**, **D**, and **F,** at least 50 cells were randomly selected from each group to score for aggresomes. Data are mean ± SEM of three independent experiments. *P < 0.05, **P < 0.01

Next, we explored whether SQSTM1 S403 phosphorylation influences the proteasome inhibition-induced aggresome generation of ubiquitinated proteins. According to previous research, the rate of aggresome production in Hela cells was slower than AD293 and A375 cells [6, 20]. Intriguingly, we discovered that blocking SQSTM1 S403 phosphorylation with a CK2 inhibitor promoted the aggresome synthesis of ubiquitinated proteins in Hela cells (Fig. 2A, B), as evidenced by K48-linked polyubiquitinated proteins (UB-K48), a well mark of proteasome inhibition-induced aggresome [6, 20, 29]. Besides, we also observed the SQSTM1 S403A overexpression significantly enhanced the aggresome formation compared to SQSTM1 WT or SQSTM1 S403E (Fig. 2C, D). In SQSTM1 knockout cells that re-expressed wildtype or mutant SQSTM1, we discovered that re-expressing S403E decreased aggresome formation during proteasome suppression (Fig. 2E, F). These data indicate that non-phosphorylation of SQSTM S403 is necessary for aggresome formation during proteasome suppression.

### Phosphorylation of SQSTM1 S403 promotes its autophagic sequestration

**Fig. 3:**
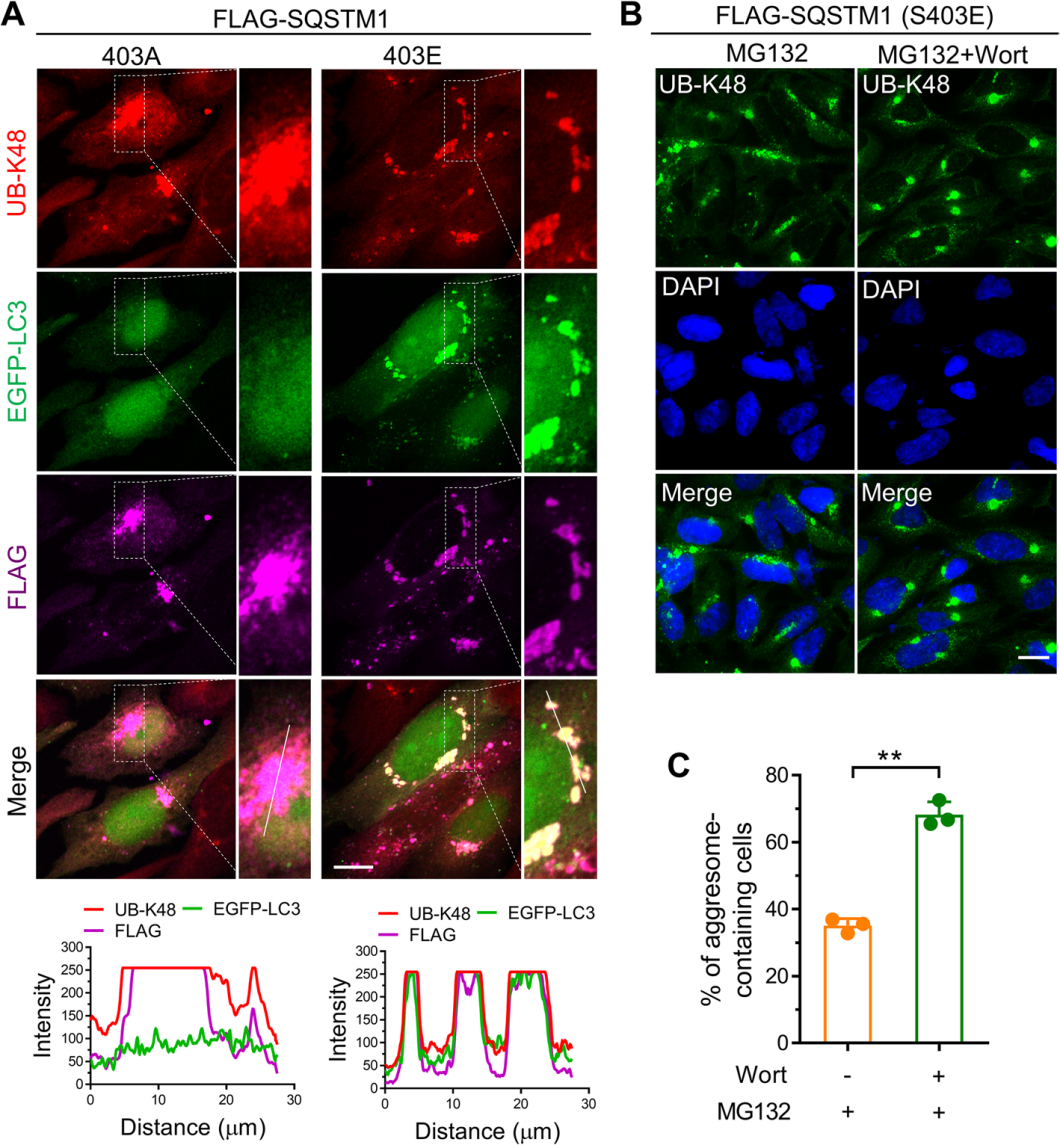
Phosphorylation of SQSTM1 S403 promotes its autophagic sequestration. **A** HeLa cells stably expressing EGFP-LC3B were transfected with plasmids expressing FLAG-SQSTM1(S403A) or FLAG-SQSTM1(S403E) for 24 h, and then treated with MG132 (1 μM) for 14 h. The cells were then fixed and analyzed by immunostaining with anti-UB-k48 (red) and anti-FLAG (meganta) antibodies. Scale bar: 10 μm. **B** SQSTM1 knockout AD293 cells stably expressing FLAG-SQSTM1(S403E) were treated with 1 μM MG132 alone or combined with Wortmannin (5 μM) for 14 h. The aggresome formation was analyzed by immunostaining with anti-UB-K48 antibodies. Nuclei were stained with DAPI (blue). Scale bar: 20 μm. **C** Quantitative analysis of results in **B**. At least 50 cells were randomly selected from each group to score for aggresomes. Data are mean ± SEM of three independent experiments. *P < 0.05, **P < 0.01

According to previous work, autophagic sequestration of SQSTM1 prevented the formation of ubiquitinated protein aggresomes [20]. We assume that S403 phosphorylation may exacerbate the autophagic sequestration of SQSTM1, which suppresses the aggresome formation. Therefore, we examined the co-localization of mutant SQSTM1 and autophagosome and found that GFP-LC3B, a well marker of autophagosome, robustly co-localized with SQSTM1 S403E and UB-K48 in micro-aggregates (Fig. 3A). In contrast, the GFP-LC3B signals were weak in SQSTM1 S403A and UB-K48-positive aggresomes (Fig. 3A). Additionally, we discovered that Wortmannin, an inhibitor of autophagy initiation, can restore SQSTM1 S403E-induced defection of aggresome formation (Fig. 3B, C). These findings indicate that SQSTM1 S403 phosphorylation may interfere with the proteasome inhibition-induced aggresome formation by aggravating the autophagic sequestration of SQSTM1 and ubiquitinated proteins.

### Phosphorylation of SQSTM1 T269/S272 inhibits the S403 phosphorylation during proteasome inhibition

**Fig. 4:**
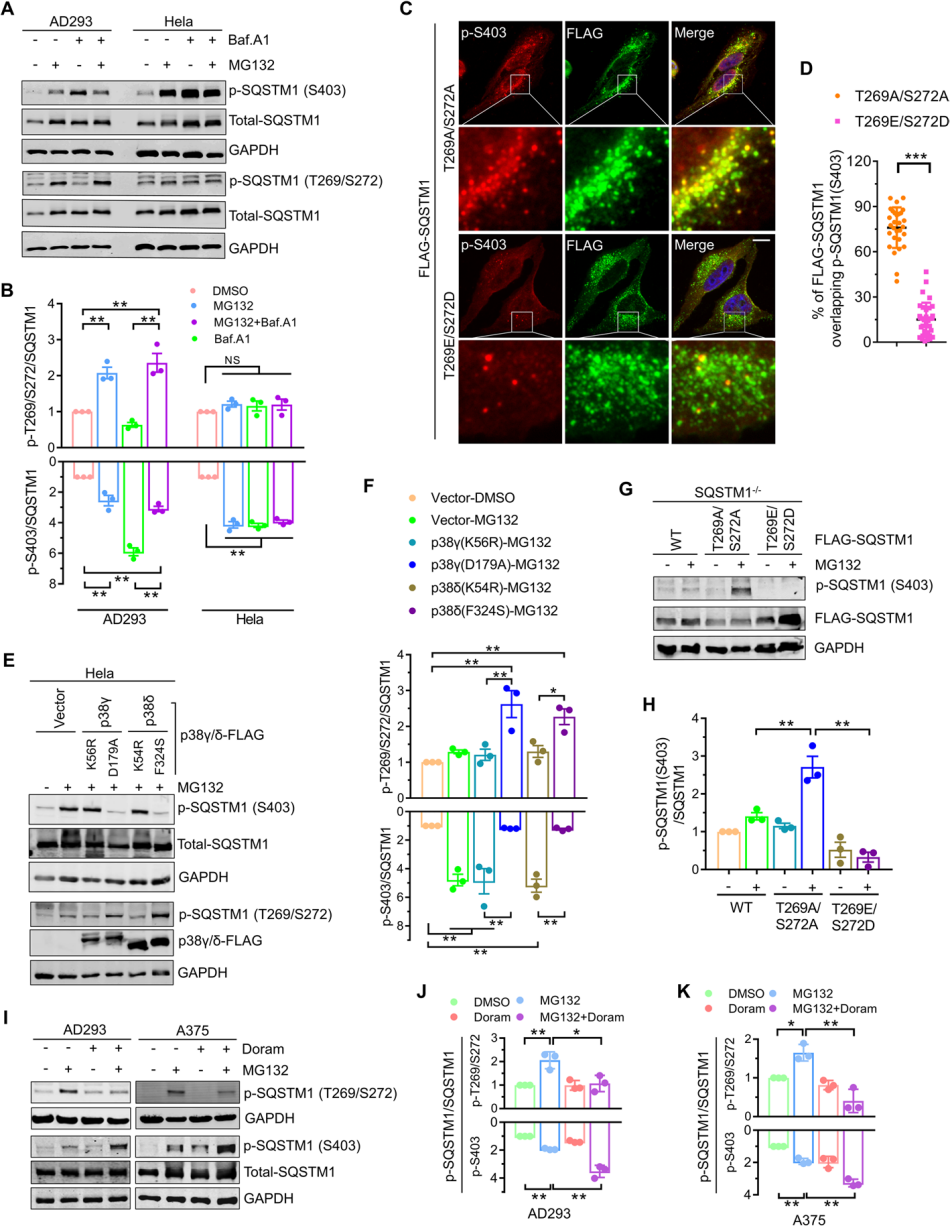
Phosphorylation of SQSTM1 T269/S272 inhibits the S403 phosphorylation during proteasome inhibition. **A** AD293 and Hela cells were treated with MG132 (2 μM), and Bafilomycin A1 (25 nM), alone or in combination for 14 h. The whole-cell lysates were subjected to western blot analysis with indicated antibodies. **B** Quantitative analysis of results in **A**. **C** HeLa cells were transfected with plasmids expressing FLAG-SQSTM1(T269A/S272A) or FLAG-SQSTM1(T269E/S272D) for 24 h, and then treated with MG132 (1 μM) and Bafilomycin A1 (25 nM) for 14 h. The cells were then fixed and analyzed by immunostaining with phospho-SQSTM1 (S403)-specific antibodies (Red) and anti-FLAG (Meganta) antibodies. Nuclei were stained with DAPI (blue). Scale bar: 10 μm. **D** Quantitative analysis of results in **C**, at least 25 cells from three independent experiments were scored for each group. **E** HeLa cells were transfected with plasmids expressing p38γ or p38δ mutants for 24 h, and then treated with MG132 (2 μM) for 14 h. The whole-cell lysates were subjected to western blot analysis with indicated antibodies. **F** Quantitative analysis of results in **E**. **G** SQSTM1 knockout AD293 cells stably expressing indicated constructs were treated with or without MG132 (2 μM) for 14 h. The whole-cell lysates were subjected to western blot analysis with indicated antibodies. **H** Quantitative analysis of results in **G**. **I** AD293 cells and A375 cells were treated with MG132 (2 μM), and Doramapimod (50 μM), alone or in combination for 14 h. The whole-cell lysates were subjected to western blot analysis with indicated antibodies. **J, K** Quantitative analysis of results in **I**. Data are mean ± SEM of three independent experiments; *P < 0.05, **P < 0.01, ***P < 0.001, *NS* no significance

Our previous studies have revealed that compared to AD293 cells and A375 cells, the SQSTM1 T269/S272 phosphorylation was limited in Hela cells due to the much lower expression of p38γ/δ that can phosphorylate SQSTM1 at T269/S272 under proteasome inhibition [6, 20]. Therefore, we speculate that the failure of proteasome inhibitor-induced suppression of SQSTM1 S403 phosphorylation in Hela cells may be caused by the ineffective SQSTM1 T269/S272 phosphorylation. To test this hypothesis, we first examined the phosphorylation levels of these sites in AD293 cells and Hela cells after being treated with proteasome inhibitors and autophagy blockers. As shown in Fig. 4A, B, when proteasome was suppressed alone or with Bafilomycin A1 in AD293 cells, the robust signal of T269/S272 phosphorylation matched the weak signal of S403 phosphorylation. Contrarily, in Hela cells, the phosphorylation level of T269/S272 did not change significantly; the phosphorylation of S403 remained at a high level, regardless of inhibition of proteasome alone or/and blocking autophagy (Fig. 4A, B). These results implicate SQSTM1 T269/S272 phosphorylation may inhibit its S403 phosphorylation. Next, we overexpressed SQSTM1 T269A/S272A and SQSTM1 T269E/S272D in Hela cells and immunofluorescence revealed the mutants' S403 site phosphorylation. We observed that after inhibiting proteasome activity and autophagic flux, the signal of S403 phosphorylation in the T269E/S272D mutant was significantly reduced compared with that in T269A/S272A mutant (Fig. 4C, D). In addition, we found overexpressed constitutively active mutants, p38γ (D179A) and p38δ (F324S), which could phosphorylate SQSTM1 T269/S272 in response to proteasome inhibition [6, 20], can significantly enhance the phosphorylation of SQSTM1 T269/S272 and inhibit the phosphorylation of SQSTM1 S403 during proteasome inhibition, rather than the kinase-dead mutants, p38γ (K56R) and p38δ (K54R) (Fig. 4E, F). SQSTM1 mutants re-expressed in SQSTM1 knockout cells were examined for S403 phosphorylation to confirm the effect of SQSTM1 T269/S272 phosphorylation. As shown in Fig. 4G, H, compared with the wildtype SQSTM1 and T269E/S272D mutants, the phosphorylation level of S403 in the T269A/S272A mutant was significantly increased during proteasome inhibition. Besides, we also observed that Doramapimod (Doram), a p38γ/p38δ inhibitor, can enhance the phosphorylation of S403 in AD293 and A375 cells under proteasome inhibition (Fig. 4I–K). These results indicate that proteasome inhibition induced SQSTM1 T269/S272 phosphorylation suppresses its S403 phosphorylation.

### Suppressing S403 phosphorylation rescues the defective aggresome formation caused by unphosphorylated SQSTM1 (T269/S272)

**Fig. 5:**
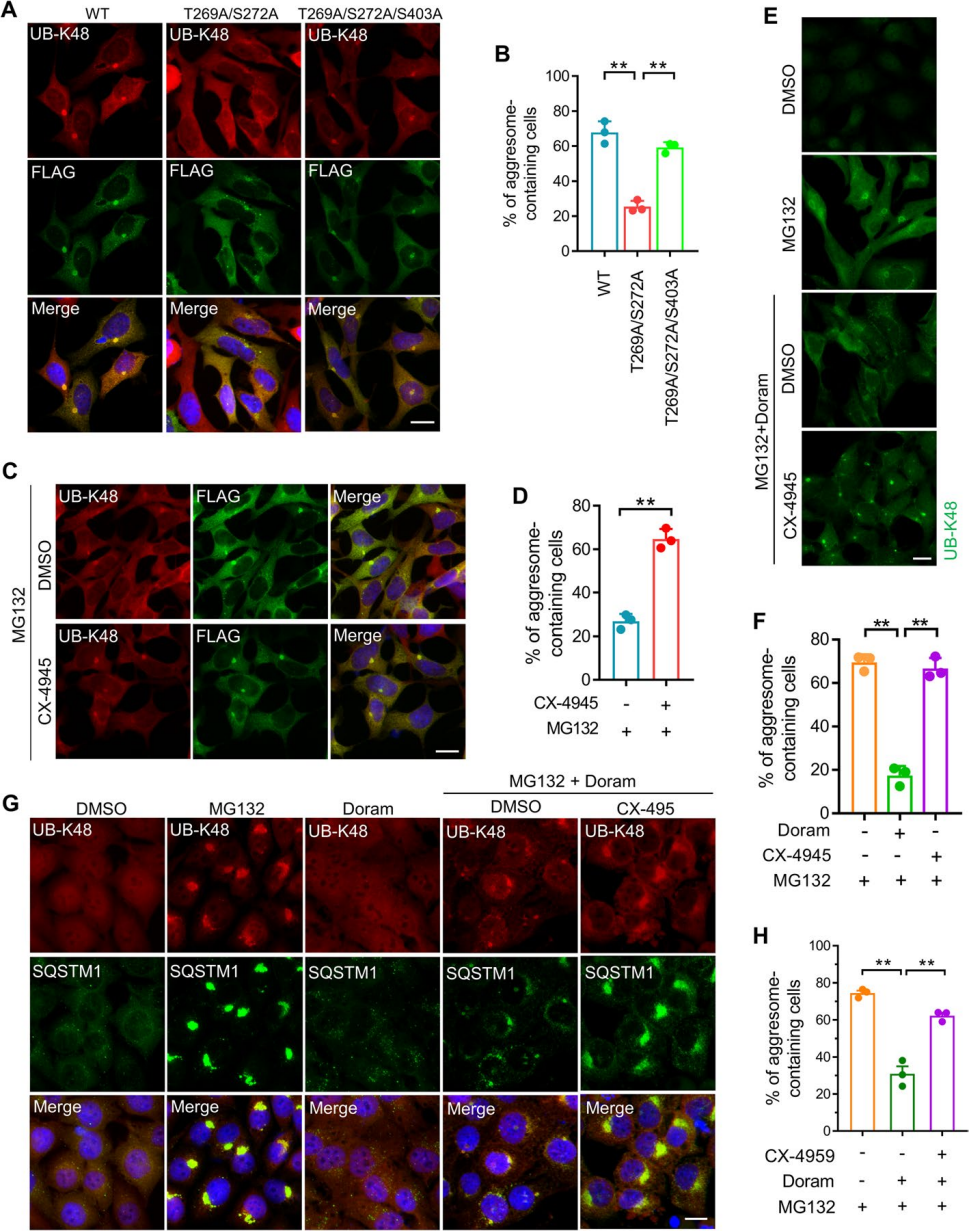
Suppressing S403 phosphorylation rescues the defective aggresome formation caused by unphosphorylated SQSTM1 (T269/S272). **A** SQSTM1 knockout AD293 cells stably expressing FLAG-SQSTM1(WT), FLAG-SQSTM1(T269A/S272A), or FLAG-SQSTM1(T269A/S272A/S403A) were treated with 1 μM MG132 for 14 h. The aggresome formation was analyzed by immunostaining with anti-UB-K48 (Red) and anti-FLAG (Green) antibodies. Nuclei were stained with DAPI (blue). Scale bar: 20 μm. **B** Quantitative analysis of results in **A**. **C** SQSTM1 knockout AD293 cells stably expressing FLAG-SQSTM1(T269A/S272A) were treated with 1 μM MG132 alone or combined with CX-4945 (5 μM) for 14 h. The aggresome formation was analyzed by immunostaining with anti-UB-K48 (Red) and anti-FLAG (Green) antibodies. Nuclei were stained with DAPI (blue). Scale bar: 20 μm. **D** Quantitative analysis of results in **C**. **E** AD293 cells were treated with MG132 (1 μM), Doramapimod (50 μM), and CX-4945 (5 μM), alone or in combination for 14 h. The aggresome formation was analyzed by immunostaining with anti-UB-K48 (Green) antibodies. Scale bar: 20 μm. **F** Quantitative analysis of results in **E**. **G** A375 cells were treated with MG132 (1 μM), Doramapimod (50 μM), and CX-4945 (5 μM), alone or in combination for 14 h. The aggresome formation was analyzed by immunostaining with anti-UB-K48 (Red) and anti-SQSTM1 (Green) antibodies. Nuclei were stained with DAPI (blue). Scale bar: 20 μm. **H** Quantitative analysis of results in **G**. For quantitative analysis of aggresome formation, at least 50 cells were randomly selected from each group to score for aggresomes. Data are mean ± SEM of three independent experiments. **P < 0.01

We predicted that SQSTM1 S403 phosphorylation would limit the aggresome formation of ubiquitinated proteins and suppress T269/S272 phosphorylation-enhanced aggresome formation. Next, we examined the impact of S403 phosphorylation on T269/S272 phosphorylation-regulated aggresome production. First, we discovered that a mutation of S403A can repair the impairment in the aggresome formation of ubiquitinated proteins caused by the T269A/S272A mutant (Fig. 5A, B). We also discovered that inhibiting CK2 can rescue this impairment (Fig. 5C, D). Furthermore, we studied the effect of CK2 inhibition on the defection of aggresome formation mediated by Doram and showed that the defection may be successfully reversed by CK2 inhibitors in AD293 and A375 cells (Fig. 5E–H). Inhibition of S403 phosphorylation appears to be the crucial mechanism for T269/S272 phosphorylation to increase the development of ubiquitinated protein aggresomes in response to proteasome inhibition.

### Suppressing S403 phosphorylation protects cells from proteasome inhibition-induced cell death

**Fig. 6:**
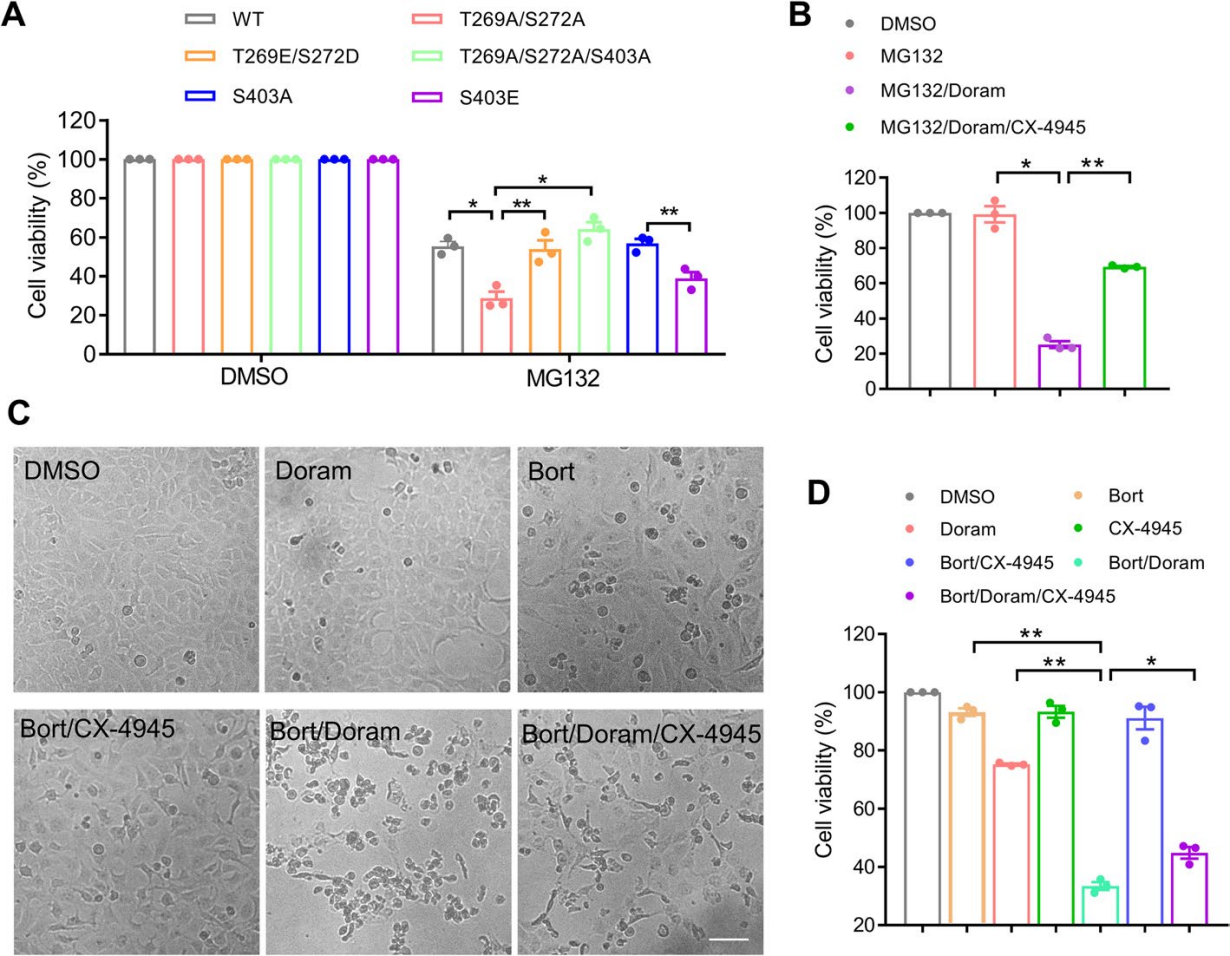
Suppressing S403 phosphorylation protects cells from proteasome inhibition-induced cell death. **A** SQSTM1 knockout AD293 cells stably re-expressing FLAG-SQSTM1(WT or mutants), were treated with 2 μM MG132 for 36, and then examined the cell viability with CCK-8 assay. **B** AD293 cells were treated with MG132 (1 μM), Doramapimod (25 μM), and CX-4945 (1 μM), alone or in combination for 24 h, the cell viability was examined with CCK-8 assay. **C, D** AD293 cells were treated with Bortezomib (20 nM), Doramapimod (25 μM), and CX-4945 (1 μM), alone or in combination for 24 h, and then the images were acquired by brightfield microscopy (**C**), the cell viability analyzed with CCK-8 assay (**D**). Scale bar: 100 μm. For **A**, **B** and **D**, data are mean ± SEM of three independent experiments. *P < 0.05, **P < 0.01

Since aggresome formation protects against cell death caused by proteasome inhibition, we wondered if blocking S403 phosphorylation might protect cells against proteasome inhibitor-induced cell death. To investigate this, we first studied the effect of SQSTM1 mutants on the cell viability of SQSTM1 knockout cells after being treated with proteasome inhibitors. Figure 6A shows that the S403E mutant caused more cell damage than S403A. Besides, we also found S403A mutation could decrease the cell damage aggravated by T269A/S272A mutation (Fig. 6A). Secondly, as the suppressor of S403 phosphorylation, CX-4945 could reverse the damage of AD293 cells aggravated by Doram (Fig. 6B). We also found that CX-4945 reduces cell damage caused by Bortezomib (Fig. 6C, D), another proteasome inhibitor, combined with Doram. These results suggest that inhibition of SQSTM1 S403 phosphorylation is conducive to cell defense against proteotoxic crisis during proteasome inhibition.

## Discussion

**Fig.7:**
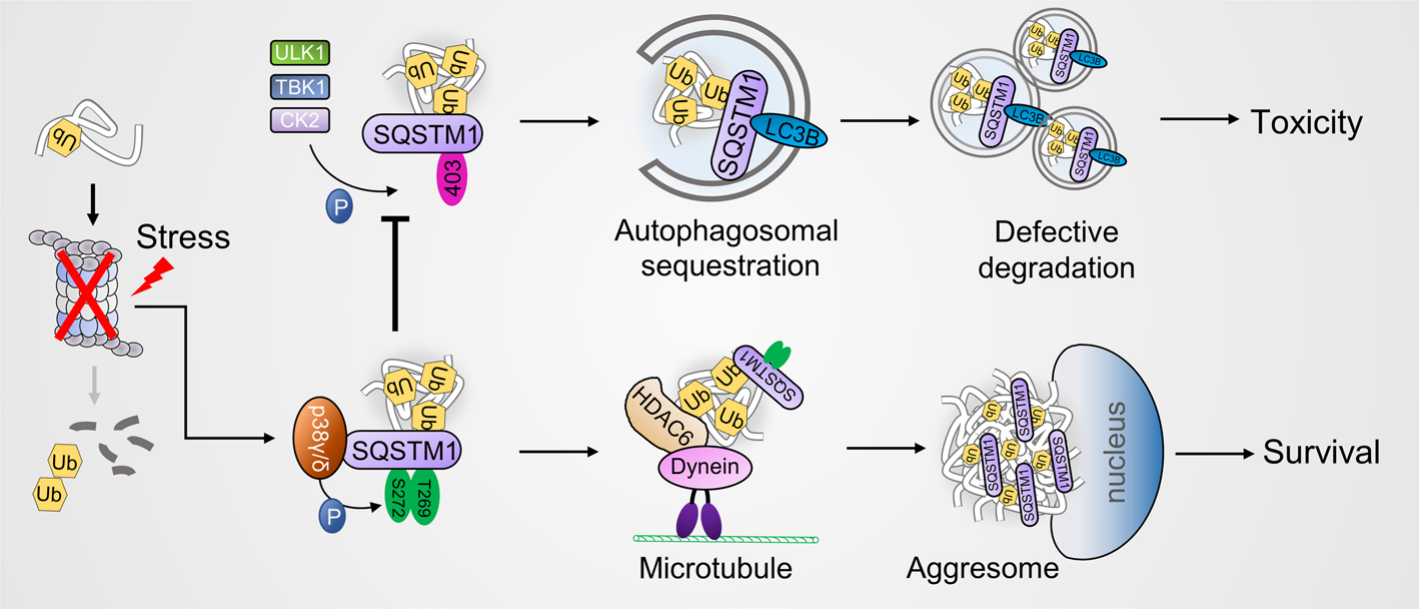
Model depicting proteasome inhibition-induced SQSTM1 T269/S272 phosphorylation inhibits its S403 phosphorylation and promotes the aggresome formation of ubiquitinated proteins

Phosphorylation alters SQSTM1's biological function. The S403 phosphorylation can enhance the affinity of SQSTM1 to ubiquitinated proteins and promote their degradation by aggrephagy [25]. However, the effect of SQSTM1 S403 phosphorylation on the ubiquitinated protein-associated aggresome formation and aggrephagy during proteasome inhibition is unclear. Herein, we reported that S403 phosphorylation does not increase ubiquitinated protein aggrephagy during proteasome suppression. Contrarily, it disrupts the aggresome formation. Besides, the SQSTM1 T269/S272 phosphorylation induced by proteasome inhibitors could suppress S403 phosphorylation, promoting aggresome formation and reducing the proteotoxic crisis (Fig. 7).

Proteasome inhibition eliminates misfolded protein-related proteotoxic crises in two steps. One is that when proteasome activity is inhibited, misfolded proteins are sequestrated into aggresome, considered the critical protective mechanism in cells at the early stage of proteasome inhibition [20, 28]. Aggresome development reduces cytoplasmic protein toxicity and misfolded protein content. Misfolded protein aggregation is a double-edged sword. If the misfolded protein in the aggresome cannot be cleared, it will aggravate cell damage [30–32]. Therefore, clearance of aggresomal misfolded proteins is required for further cellular protection against proteasome inhibition. However, proteasome inhibition increases autophagy later than aggresome production [20, 28]. In this process, it is still controversial whether subsequently enhanced autophagy attenuates the proteotoxic crisis of cells or aggravates cell damage [20, 28]. One possible reason is that the autophagic clearance of aggresomal ubiquitinated proteins depends on the normal proteasome. Indeed, in the proteasome inhibitor washout experiment, aggresomal ubiquitinated proteins can be efficiently degraded through the autophagy system rather than in the micro-aggregates [20, 33, 34]. Therefore, the imbalance between misfolded protein aggregation and degradation may be the fundamental pathogeny of misfolded protein-related disorders like neurodegenerative disease [35] and cardiomyopathy [36].

SQSTM1 S403 phosphorylation is an important regulatory point in the aggrephagy of ubiquitinated proteins. However, S403 phosphorylation enhances the autophagic breakdown of ubiquitinated proteins, Shinrye Lee et al. reported that in neuronal cells, S403A mutants can decrease cell damage by enhancing the autophagic clearance of insoluble ubiquitinated proteins during UPS dysfunction [37]. Moreover, Matsumoto et al. also found that S403-phosphorylated SQSTM1 was mainly localized to micro-aggregates in the cytoplasm rather than aggresome in aggresome-formed neuronal cells [38]. Interestingly, proteasome inhibition-independent aggresomes can sequester phosphorylated SQSTM1 S403 as well [38]. These results suggest different regulatory mechanisms of SQSTM1 in proteasome inhibition-dependent and -independent aggresome formation. SQSTM1 S403 phosphorylation increased micro-aggregates of ubiquitinated proteins rather than aggresomes after proteasome suppression. Besides, we also found that blocking the initiation of autophagy could rescue the defection of S403 phosphorylation-induced defection of aggresome formation, indicating that S403 phosphorylated SQSTM1 may disrupt the aggresome formation of ubiquitinated protein through promoting its autophagic sequestration.

We previously found that SQSTM1 T269/S272 phosphorylation during proteasome inhibition prevents autophagic sequestration and promotes the aggresome formation of ubiquitinated protein by weakening its binding to LC3B [20]. Here, we found that T269/S272 phosphorylation can also inhibit the phosphorylation of S403. One possibility is that the conformational switching of SQSTM1 induced by T279/S272 phosphorylation might inhibit the binding and modification of kinases responsible for S403 phosphorylation to SQSTM1. Further study will address this possibility. Recent investigations on liquid–liquid phase separation-formed SQSTM1-related biomolecular condensates may offer a novel explanation. Yu et al. reported that phosphorylation of the S403 can promote SQSTM1 to bind ubiquitinated proteins and form liquid–liquid phase-separated condensates, facilitating its autophagic degradation [39]. Seo Hyeong Park et al. found that proteasome inhibition-induced aggresomes are less dynamic hydrogel-like condensates than liquid droplets [40]. Combined with the results reported here, these findings suggest that phosphorylation at different sites of SQSTM1 may mediate condensates with different characteristics, in which the molecular mechanisms for the modification of SQSTM1 phosphorylation are different. Additionally, we observed that K63-linked polyubiquitinated proteins (UB-K63), which also involved in SQSTM1-associated aggrephagy [22], did not form aggregate or aggresome as significantly as UB-K48 does during proteasome inhibition (data not shown), suggesting that the behavior of these two types of ubiquitinated proteins diverges when the proteasome is inhibited. One plausible explanation for this is that the type of ubiquitin ligation determines the characteristics of the aggregated ubiquitinated protein aggregate/aggresome, with UB-K48 formed aggregates being conducive to transportation and aggresome formation within the cytoplasm.

In conclusion, SQSTM1 T269/S272 phosphorylation inhibits S403 phosphorylation during proteasome inhibition, boosting the aggresome formation of ubiquitinated protein and shielding cells from proteotoxic crisis. Our research showed that SQSTM1 phosphorylation links autophagic degradation and aggresome production of ubiquitinated proteins.

### Supplementary Information

**Additional file 1: Table S1**. Sequences of the primers used for ORF amplification. **Table S2** Antibody information.

## Data Availability

All relevant data are contained within the main manuscript or supplemental information. Please email zhangchenliang@wchscu.cn with requests for raw data or reagents.

## Abbreviations

SQSTM1: Sequestosome 1
HSPs: Heat shock proteins
CHIP: Carboxyl-terminal of Hsp70/Hsp90 interacting protein
BAG3: Bcl-2-associated athanogene 3
PKM2: M2 isoform of pyruvate kinase
MTOC: Microtubule organizing center
ER: Endoplasmic reticulum
CK2: Casein kinase 2
TBK1: TANK-binding kinase 1
ULK1: UNC-51-like kinase 1
UB-K48: K48-linked polyubiquitinated proteins
UB-K63: K63-linked polyubiquitinated proteins
