# Supplementary material for: Inhibition of SQSTM1 S403 phosphorylation facilitates the aggresome formation of ubiquitinated proteins during proteasome dysfunction
Source: Cell Mol Biol Lett. 2023 Oct 24;28:85. doi: 10.1186/s11658-023-00500-6 (PMC10594750; doi:10.1186/s11658-023-00500-6)
Supplement: Supplementary file 1 — Additional file 1: Table S1. Sequences of the primers used for ORF amplification. Table S2 Antibody information. [file 11658_2023_500_MOESM1_ESM.docx]

**Inhibition of SQSTM1 S403 phosphorylation facilitates the aggresome formation of ubiquitinated proteins during proteasome dysfunction**

Chenliang Zhang^1, *^, Chen Huang^2^, YiChun Duan^2^, Liping Li^3^

^1^Division of Abdominal Cancer, Department of Medical Oncology, Cancer Center and Laboratory of Molecular Targeted Therapy in Oncology, West China Hospital, Sichuan University, Chengdu, 610041, Sichuan Province, China;

^2^Division of Abdominal Cancer, Department of Medical Oncology, Cancer Center, West China Hospital, Sichuan University, Chengdu, 610041, Sichuan Province, China;

^3^Department of Pharmacy, Chengdu Fifth People's Hospital, Chengdu, 611130, Sichuan Province, China.

^*^For correspondence: Chenliang Zhang, zhangchenliang@wchscu.cn

**Key words:** SQSTM1; proteasome inhibition; aggresome; cell death

**Running title:** SQSTM1 S403 phosphorylation inhibits aggresome formation

| **Table S1. Sequences of the primers used for ORF amplification** | | |
| --- | --- | --- |
| **DNA Construct** | **PCRed DNA Fragment** | **Primer Name and Sequence (5'-3')** |
| SQSTM1-S403A | SQSTM1-N | sqstm1EcoR-f：CGATAAAGGATCCGAATTCatggcgtcgctcaccgtgaaggcc |
|  |  | sqstm1-403A-r：catcagagaagcccatAGCcagcat |
|  | SQSTM1-C | sqstm1-403A-f：atgctgGCTatgggcttctctgatg |
|  |  | sqstm1Xho-r：ATGGGTATCTAGACTCGAGtcacaacggcgggggatgctttgaat |
| SQSTM1-S403E | SQSTM1-N | sqstm1EcoR-f：CGATAAAGGATCCGAATTCatggcgtcgctcaccgtgaaggcc |
|  |  | sqstm1-403E-r：catcagagaagcccatGAAcagcat |
|  | SQSTM1-C | sqstm1-403E-f：atgctgTTCatgggcttctctgatg |
|  |  | sqstm1Xho-r：ATGGGTATCTAGACTCGAGtcacaacggcgggggatgctttgaat |
| p38γ-K56R | p38γ-N | p38γHind-f：CCGCTAGCCTTAAGCTTGCCACCatgagctctccgccgcccgcccgc |
|  |  | p38γ-56R-r：gccgatacagcttCCGgatggccac |
|  | p38γ-C | p38γ-56R-f：gtggccatcCGGaagctgtatcggc |
|  |  | p38γXba-f：TCATCCTTGTAATCTCTAGAcagaggcgtctccttggagacc |
| p38γ-D179A | p38γ-N | p38γHind-f：CCGCTAGCCTTAAGCTTGCCACCatgagctctccgccgcccgcccgc |
|  |  | p38γ-179A-r：gtcatctcactAGCtgcctgcctgg |
|  | p38γ-C | p38γ-179A-f：ccaggcaggcaGCTagtgagatgac |
|  |  | p38γXba-f：TCATCCTTGTAATCTCTAGAcagaggcgtctccttggagacc |

| Table S2 Antibody information | | | |  |
| --- | --- | --- | --- | --- |
| Antibody | Company | Cat # | Dilution |  |
| UB-K48 | Millipore | 05-1307 | IF: 1:300 |  |
| ubiquitin | Abcam | ab134953 | WB: 1:1000 |  |
| Phospho-SQSTM1 (S403)-specific antibody | Cell Signaling Technology | 39786 | WB: 1:1000 IF: 1:250 |  |
| Phospho-SQSTM1 (Thr269/Ser272) | Phosphosolutions | P196-269 | WB: 1:1000 |  |
| SQSTM1 | Proteintech, | 18420-1-AP | WB: 1:1000 |  |
| SQSTM1 | Santa Cruz Biotechnology | sc-28359 | WB: 1:2000 IF: 1:300 |  |
| LC3B | Cell Signaling Technology | 3868 | WB: 1:1000 |  |
| FLAG tag | Prospec | ANT-146-b | WB: 1:2000 IF: 1:200 |  |
| Myc Tag | Biolegend | MMS-150R | WB: 1:2000 |  |
| GAPDH | Zen Bioscience | 200306 | WB: 1:5000 |  |
| β-actin | Zen Bioscience | 200068-6D7 | WB: 1:5000 |  |
| Dylight 680/800-conjugated secondary antibodies | Thermo Fisher Scientific | A28183, A27042, 35518 | WB: 1:10000 |  |
|  |  |  |  |  |
|  |  |  |  |  |
| Alexa Flour 488- or 568-conjugated secondary antibodies | Thermo Fisher Scientific | A11034, A11029, A11031, A11036 | IF: 1:1000 |  |
|  |  |  |  |  |
|  |  |  |  |  |
|  |  |  |  |  |
